# Applying $f_{4}$-statistics and admixture graphs: theory and examples

**DOI:** 10.1111/1755-0998.13230

**Published:** 2020-08-19

**Authors:** Mark Lipson

**Affiliations:** 1 Department of Genetics, Harvard Medical School, Boston, MA 02115, USA; 2 Department of Human Evolutionary Biology, Harvard University, Cambridge, MA 02138, USA

**Keywords:** *f*-statistics, admixture graphs, admixture, parameter estimation

## Abstract

A popular approach to learning about admixture from population genetic data is by computing the allele-sharing summary statistics known as f-statistics. Compared to some methods in population genetics, f-statistics are relatively simple, but interpreting them can still be complicated at times. In addition, f-statistics can be used to build admixture graphs (multi-population trees allowing for admixture events), which provide more explicit and thorough modeling capabilities but are correspondingly more complex to work with. Here, I discuss some of these issues to provide users of these tools with a basic guide for protocols and procedures. My focus is on the kinds of conclusions that can or cannot be drawn from the results of $f_{4}$-statistics and admixture graphs, illustrated with real-world examples involving human populations.

## Introduction

f-statistics (Reich et al., 2009; Patterson et al., 2012) are a widely used toolkit for making inferences about phylogeny and admixture from population genetic data, particularly in humans. The statistics measure correlations in allele frequencies among sets of two, three, or four populations. Observed values reflect degrees of shared ancestry and can serve as a means for testing hypotheses regarding population split orders and past gene flow events under historical models.

As compared to some other common methods in population genetics, f-statistics are quite simple and flexible, but interpreting them is not always straightforward. Additionally, one of the primary applications of f-statistics is in building admixture graphs (i.e., phylogenetic trees augmented with admixture events) with more than four populations, which introduces a greater level of complexity. In this note, I hope to clarify some of these potential difficulties and provide a range of tips for practitioners. Some of the topics have been addressed previously but are covered here as well for the sake of completeness.

## f-statistics and admixture

### Basic definitions and properties

More complete introductions to f-statistics have been published elsewhere (Reich et al., 2009; Patterson et al., 2012; Lipson et al., 2013; Peter, 2016; Soraggi and Wiuf, 2019), but the following are some basics that are used in other sections of the paper. The most general definition is that of the $f_{4}$-statistic $f_{4}(A,B;C,D)$, which measures the average correlation in allele frequency differences between (i) populations A and B and (ii) populations C and D (i.e., $\left(p_{A}-p_{B}\right)*\left(p_{C}-p_{D}\right)$, for allele frequencies p, typically averaged over many biallelic single-nucleotide polymorphisms [SNPs]). This $f_{4}$-statistic is the same as the (perhaps more familiar) D-statistic up to a normalization factor. If the four populations are related by the (unrooted) phylogeny ((A,B),(C,D)), then the expected value of $f_{4}(A,\;B;\;C,\;D)$ will be zero, while the expected values of $f_{4}(A,\;C;\;B,\;D)$ and $f_{4}(A,\;D;\;B,\;C)$ will be positive. (When I refer to expectations of f-statistics, I mean with respect to the random noise in real data—typically assumed to be normally distributed—caused by sampling finite numbers of independent SNPs and individuals.) Simple algebra shows that

$$\begin{array}{l} f_{4}\left(A,B;C,D\right)=f_{4}\left(C,D;A,B\right),\\ f_{4}(A,B;C,D)=-f_{4}(B,A;C,D)=-f_{4}(A,B;D,C)\\ f_{4}\left(A,B;C,D\right)=f_{4}\left(A,C;B,D\right)+f_{4}\left(A,D;C,B\right). \end{array},$$

The other two are of basic definitions the $f_{2}$- and $f_{3}$-statistics, which can be formulated as $f_{2}(A,B)=f_{4}(A,B;A,B)$ and $f_{3}(A;B,C)=f_{4}(A,B;A,C)$.

The most important usage for f-statistics is in the context of admixture. If a population C has a mixture of ancestry derived from sources $C^{\prime }$ and $C^{\prime \prime }$ in proportions α and (1−α), then in expectation,

$$f_{4}\left(A,B;C,D\right)=\alpha f_{4}\left(A,B;C^{\prime },D\right)+\left(1-\alpha \right)f_{4}\left(A,B;C^{\prime \prime },D\right).$$

**Figure 1.**
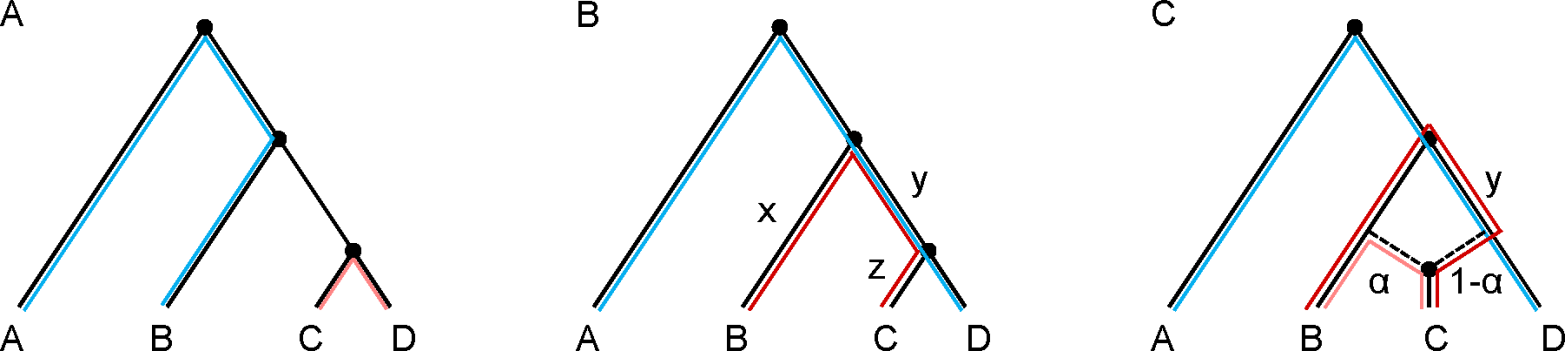
Expected values of $f_{4}$-statistics under specified admixture graph models. (A) The expected value of $f_{4}(A,B;C,D)$ is given by the intersection between the path from A to B with the path from C to D. Under the model shown, $E\left[f_{4}(A,B;C,D)\right]=0$. (B) The expected value of $f_{4}(A,D;B,C)$ is given by the intersection between the path from A to D with the path from B to C. Under the model shown, $E\left[f_{4}(A,D;B,C)\right]=y$. (C) With population C admixed, the path from B to C can be decomposed into two components. Under the model shown, with a proportion of α B-related ancestry and 1−α D-related ancestry, the former yields a path (lighter red) that has a weight of α but does not intersect the path from A to D, while the latter yields a path (darker red) that has a weight of 1−α and intersects the path from A to D over the branch with length y. In total, $E\left[f_{4}(A,D;B,C)\right]=(1-\alpha )y$.

Expected values of f-statistics can be visualized in terms of overlapping paths in an admixture graph (Fig. 1; see also Patterson et al. (2012); Peter (2016); Soraggi and Wiuf (2019)). In the case of admixture, the above equation can be used to derive the expectation in terms of a weighted sum of path-overlaps involving each source (Fig. 1C). Thus, if C is admixed, the typical expected value of $f_{4}(A,B;C,D)$ will be a branch length times a mixture proportion (Fig. 1C).

Unlike $F_{ST}$ (and normalized D-statistics, at least approximately), the values of f-statistics (including branch lengths in admixture graphs that are defined in f-statistic units, as in Fig. 1) depend on the absolute allele frequencies of the SNPs used to calculate them (cf. Lipson et al. (2013)). For example, adding fixed sites to the SNP set will shrink f-statistics toward zero. As a result, when comparing multiple f-statistics, it is important that each one should be computed on the same set of SNPs (or as similar as possible). In applications involving ancient DNA, where missing data is common, I typically make the assumption that the SNPs covered for each individual or population are a random subset with respect to allele frequency. By contrast, comparisons across different genotyping arrays are likely to be biased.

### Interpreting non-zero $f_{4}$-statistics

**Figure 2.**
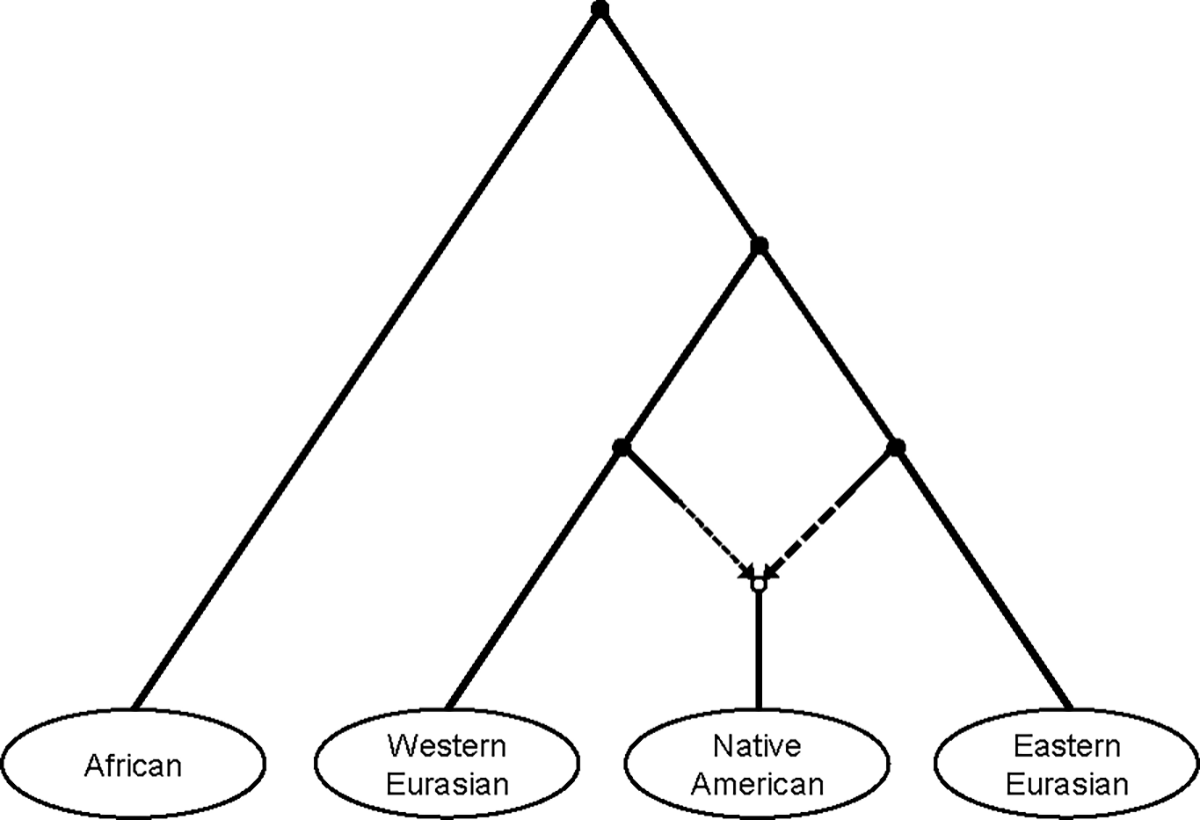
Major human lineages used for examples in the paper, represented by Baka (African), French (western Eurasian), Mixe (Native American), and Han (eastern Eurasian). Setting aside other complexities in the histories of these populations, the admixture event being modeled involves eastern and western Eurasian lineages contributing ancestry to Native Americans (Raghavan et al., 2014). See Figs. 3A and 5A for fitted models using this correct topology.

If a set of four populations are unadmixed relative to each other, then some permutation of them will yield an $f_{4}$-statistic of zero (in expectation), as in Fig. 1A. Equivalently, if all three permutations of $f_{4}$-statistics for a certain set of four populations are (significantly) non-zero, then at least one of the populations must be admixed; this is one of the most common signals of admixture used in the literature. In this paper, I will use the example of a quartet consisting of four present-day human populations: Mixe (from Mexico), Han Chinese, French, and Baka (hunter-gatherers from Cameroon). The common ancestral population of all Native Americans is known to have been admixed with approximately 70% ancestry from an eastern Eurasian lineage and 30% from a western Eurasian lineage (Fig. 2) (Raghavan et al., 2014). Thus, in the context of this quartet, Mixe can be modeled as admixed with ancestry related to Han (~70%) and to French (~30%). I computed the three possible $f_{4}$-statistics for the quartet and obtained significantly non-zero values, with the signs as expected based on the known history (Table 1). (These and all results in the paper are computed from previously published whole-genome sequence data (Mallick et al., 2016; Fan et al., 2019), on a set of ~1.1 million autosomal SNPs (Mathieson et al., 2015), using the implementation in ADMIXTOOLS (Patterson et al., 2012), including standard errors estimated by block jackknife.)

In this case, there is prior knowledge available about the admixture in Mixe, but in general, without additional information, the existence of such a quartet does not identify which of the four populations is admixed. Here, for example, it could also be that Han is admixed with most of its ancestry related to Mixe but a small amount related to Baka, and likewise for the other two (see further discussion in the admixture graph sections below). In real-world applications, it can also be true that more than one population is admixed, making the interpretation more complicated. Sometimes, in fact, two admixture events together can cause an $f_{4}$-statistic to be close to zero and thereby mask the signal of admixture (at first glance).

Another observation is that as depicted in Fig. 1, $f_{4}$-statistics are not only zero or non-zero but also carry quantitative information about amounts of shared drift between populations. One implication is that populations sharing more drift (i.e., yielding longer intersecting paths in an admixture graph) will have greater-magnitude $f_{4}$-statistics associated with them. For example, in the trees of Fig. 1B–C, if one replaced population D with a population $D^{\prime }$ that split halfway between D and the root of the tree, then the expected magnitude of $f_{4}\left(A,B;C,D^{\prime }\right)$ would be smaller, since the length of the shared drift branch would now be less than y. As a result, under the model in Fig. 1C, one could use the fact that $f_{4}(A,B;C,D)>f_{4}\left(A,B;C,D^{\prime }\right)$ to conclude that D is a better proxy than $D^{\prime }$ for the ancestry in C (the component with proportion 1−α). However, this procedure is complicated by the fact that if the D-related source was in fact itself admixed, with ancestry related to X and Y, then the $f_{4}$-statistic can sometimes be maximized by X or Y instead of by D, even though one would consider D to be a better proxy (Pickrell et al., 2014). It is also good to remember that if a certain signal is weak compared to the noise in the data—for example, if one were testing for admixture in C and the shared drift branch length y was short—then one may not have enough power to identify it.

Finally, f-statistics can be subject to certain kinds of biases and batch effects (to varying degrees, as with other methods) arising from SNP ascertainment, sample type and processing (ancient versus present-day, sequencing platform, etc.), and other aspects of the data, so it is important to keep such factors in mind when interpreting results. For ancient DNA data, challenges include C-to-T errors induced by postmortem deamination (Hofreiter et al., 2001), as well as short fragment lengths and (often) low coverage, which can exacerbate reference bias (Günther and Nettelblad, 2019). All of these effects can cause ancient individuals to appear artificially closely related to one another and to certain other populations (e.g., deep outgroups). In general, statistics $f_{4}(A,B;C,D)$ in which A and C share a data type and B and D share a different data type are most prone to this kind of artifact.

## Admixture graphs: modeling and inference procedure

### Fitting an admixture graph with *qpGraph*

In addition to their stand-alone usage, f-statistics can serve as a means to fit admixture graphs from allele frequency data. (Other kinds of statistics can also be used to fit admixture graphs, but I will not discuss such methods in detail here; see Discussion.) In this context, an admixture graph consists of an ordering of population splits, positions of admixture events, branch length parameters, and mixture proportions. Given the first two, the third and fourth can be inferred by solving a system of equations (linear in terms of the branch lengths) in which observed f-statistic values are matched to their expectations in terms of the model parameters. For example, one such equation for the model in Fig. 1B would be $f_{2}(B,C)=x+y+z$. With n populations, there are 3×n4 possible $f_{4}$-statistics, 3×n3 possible $f_{3}$-statistics, and n2 possible $f_{2}$-statistics, but many of these are linearly dependent; for example, $f_{4}(A,\;B;\;C,\;D)=f_{3}(A;\;B,\;D)-f_{3}(A;\;B,\;\;C)$. In fact, there are a total of n2 linearly independent f-statistic equations, or in other words, f-statistics form a vector space of dimension n2. Possible choices of basis include (1) the set of all $f_{2}$-statistics, and (2) the set of all $f_{2}$- and $f_{3}$-statistics with a given population in the first position.

The software I typically use to build admixture graphs is *qpGraph* (also referred to as ADMIXTUREGRAPH) (Patterson et al., 2012). In *qpGraph*, the user manually specifies the topology of the model, and the program then solves for the optimal values of the parameters. In theory, one might wish to search the entire space of all topologies and parameter values (for a given number of admixture events) to find the best-fitting model, but the size of the space (exponential in the number of populations) makes this impractical for larger graphs (Leppälä et al., 2017). The set of basis statistics used for fitting is the set (2) alluded to in the previous paragraph, with the first population listed in the input file as the “base” population.

In its standard mode, *qpGraph* attempts to minimize the quantity $S(G)=1/2(g-f{)}^{\prime }Q^{-1}(g-f)$, known as the “score” of the model, where f is the vector of observed basis f-statistics (of length n2), g is the vector of predicted f-statistics under the model, and Q is the (estimated) covariance matrix of the statistics. Assuming multivariate normal errors, the score gives the negative log-likelihood of the model; it measures the total amount by which the system of f-statistic equations (one for each basis statistic) fails to be satisfied, taking into account the empirical correlation among the statistics (see also the next section on fit quality). To help insure that $Q^{-1}$ does not become unstable, one can use the “diag” input parameter to add a small number (“diag: 0.0001” works well in my experience, but smaller values may be sufficient as well) to the diagonal entries of Q. The program can also be run using simple least-squares optimization without the Q matrix by specifying “lsqmode: YES,” but in this case highly correlated statistics will be treated as independent for the sake of the fitting, and the score will no longer represent a log-likelihood, both of which make the full objective function preferable. Other input parameters I typically set are “outpop: NULL” (meaning no specified outgroup population in which SNPs are required to be polymorphic) and “lambdascale: 1” (leaving the f-statistics in typical units rather than scaling into approximate $F_{ST}$). More extensive descriptions of the *qpGraph* software can be found in Patterson et al. (2012) and in the ADMIXTOOLS package repository (https://github.com/DReichLab/AdmixTools), and of the f-statistic-based admixture graph inference process more generally in Lipson et al. (2013); Leppälä et al. (2017).

By default, *qpGraph* utilizes the set of SNPs that have genotype calls for at least one individual in each population in the model. With low-coverage data (for example, in some ancient DNA applications), this can result in losing the majority of the sites in the initial data set. The program allows an option to use all SNPs instead (“allsnps: YES” or “use-allsnps: YES,” in which case each basis statistic is computed on as many sites as possible for the two or three populations involved), but this mode can give unreliable results, in particular when the base population is highly diverged from the other populations in the model. To the best of my knowledge, this effect is caused by greater absolute noise when estimating larger-magnitude basis statistics, such that the small relative fluctuations in empirical f-statistics caused by modest changes in the SNP set become substantial in the context of the admixture graph. In my own work, my preference has always been to avoid using the all-SNPs option. If this causes an undesirable loss of coverage, then the best approach given the current implementation of *qpGraph* is probably to set as the base a population that (a) is not highly diverged from the others in the model, and (b) preferably has multiple individuals with diploid data (again to reduce the magnitudes of the statistics). Research is currently underway aiming to develop an improved all-SNPs methodology.

### Parameters and constraints

**Figure 3.**
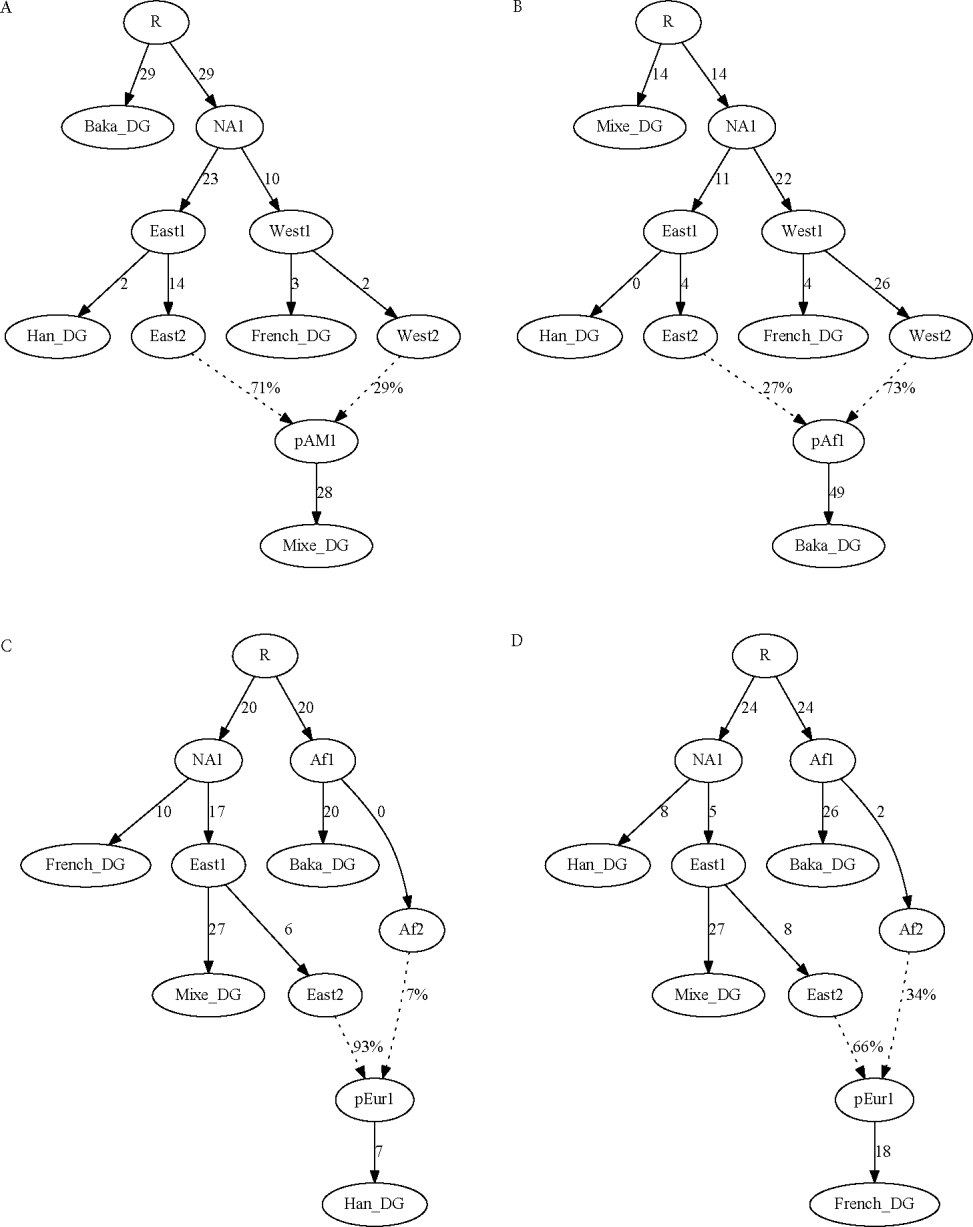
Four-population admixture graphs modeling (A) Mixe, (B) Baka, (C) Han, or (D) French as admixed. All four versions provide perfect fits to the data (exact agreement between observed and predicted f-statistics). In this and all following figures, branch lengths (in f-statistic units, multiplied by 1000) are rounded to the nearest integer.

An important consideration is whether the system of equations used to infer the parameters of an admixture graph is over- or under-determined. As mentioned above, a model with n populations has n2 linearly independent constraints (i.e., equations). In the absence of admixture, there are 2n−3 parameters, which is the number of branches in an unrooted binary tree with n leaf nodes (with the settings I have described, *qpGraph* results should not depend on where the root of a graph is specified). Converting a population from unadmixed to admixed adds two parameters: one for the mixture proportion and one for the split position of the new source of ancestry. Thus, with *a* admixture events, the total number of free parameters is 2n+2a−3. One point to note is that in the case of an admixed population with two unsampled sources (which is the typical scenario), the three branch lengths surrounding the admixture event (in Fig. 3A, from the node “East1” to “East2,” from “West1” to “West2,” and from “pAM1” to Mixe) cannot be determined individually but instead form a single compound parameter $\alpha^{2}x+(1-\alpha {)}^{2}y+z$ (where α is the mixture proportion, x and y are the branch lengths to the two corresponding sources, and z is the terminal branch length). The only exception (to my knowledge) is the case in which at least three populations are included that can be modeled as having different proportions of ancestry from the same two sources, which allows the branch lengths to be solved for individually.

Even if the inequality n2≥2n+2a−3 is satisfied for an admixture graph as a whole, there can be some parameters that are not uniquely determined because of repetition across the different equations caused by multiple populations in phylogenetically equivalent positions. Further discussion of this phenomenon can be found in the example sections below. Additionally, having sufficient constraint to estimate parameters is not entirely a yes-or-no proposition. A model can have enough populations in distinct positions to be able to estimate a mixture proportion, but if two of the populations are only slightly separated, then the precision of the estimate will generally be lower. Similarly, if one of the populations providing the constraint is itself admixed, then the power will often be reduced.

### Fit quality

To my knowledge, no absolute measure of model fit has been developed for admixture graphs, but there are several ways to evaluate how well a given model fits the data (this is an area of active study; see also Lipson and Reich (2017); Lipson et al. (2017); Leppälä et al. (2017); Flegontov et al. (2019); Shinde et al. (2019); Lipson et al. (2020)). The following discussion is tailored for *qpGraph*, but the ideas also apply more generally. First, the program returns a list of residual poorly-predicted f-statistics and their *Z*-scores (drawn from the set of all possible f-statistics, not only those in the basis), which can give a good sense for the performance of the model and some idea of which populations are responsible for the greatest inaccuracies. There is no general rule for what threshold constitutes a significantly non-zero residual; the situation is complicated because there are many statistics being tested simultaneously, but many of those are also correlated with each other.

Deviations between model predictions and the observed data can be caused either by an incorrectly specified topology or un-modeled admixture. In the first case, assuming that the program does not get stuck at a local optimum, it will try to move the populations as close as possible to their correct positions but will be constrained by the input topology. Thus, an incorrectly specified split order usually manifests as an inferred length-zero internal branch; when such branches (i.e., trifurcations) appear in the results, the order of splits should be adjusted and re-tried. (The default *qpGraph* visualization output rounds branch lengths to the nearest integer, so some non-zero-length but very short branches may initially appear as zero.) As noted in the f-statistics section above, however, one may not have sufficient power to resolve short branches, so some sets of three lineages may be found to be statistically consistent with forming a trifurcation, with all three possible split orders having similar fit quality.

In the case of un-modeled admixture, the observed deviations could potentially reflect admixture in one of multiple different populations. Often one can gain information by examining the full list of residuals and noting which populations occur repeatedly. Another approach is to remove one population from the model and see if the fit improves, although even if it does, that could imply either that the population in question had un-modeled admixture or that it provided a constraint enabling the detection of un-modeled admixture among the other populations.

The score of the final graph is also returned as an output from the program, so it can be used to compare the fit quality of different models with the same set of populations, preferring the one with the lower score. (If the equations being fit were independent, then one could apply a chi-squared test for the overall fit, but in practice they are heavily correlated. *qpGraph* returns a naive degrees of freedom count and *p*-value alongside the score, but they are not well calibrated.) As above, while this approach provides a useful heuristic, evaluating statistical significance is complicated, and I do not have a rigorous set of recommendations. One recent direction that seems promising is using the score to compare alternative models with the same populations and same number of admixture events. In that case, the score difference can be interpreted in an AIC/BIC framework, with the likelihood difference as a Bayes factor (Leppälä et al., 2017; Flegontov et al., 2019; Shinde et al., 2019). The same idea could also be applied in cases with unequal numbers of free parameters—for example, adding one admixture event and testing whether the score improvement is significant. However, defining the change in degrees of freedom is not straightforward in this situation: as noted above, a new admixture event creates two additional parameters in the model, but that does not account for whether the admixture comes from a pre-specified source or from a source that is allowed to be located anywhere in the graph. Finally, the score can additionally be used to compute confidence intervals on parameters (by considering the likelihood as a function of a single branch length or mixture proportion value), although it is worth keeping in mind that the results are model-dependent.

## Admixture graphs: examples

One of the strengths of f-statistic-based admixture graphs is that they are computationally tractable enough that programs such as *qpGraph* can accommodate a large number of populations and admixture events. Sometimes though it can be difficult to digest all of the information in large admixture graph models and to analyze their behavior. Fortunately, the main principles of admixture graph fitting can be illustrated with simpler examples, which, in particular, carry over directly to larger models by considering subsets of four and five populations.

### Four populations

The first examples I will present are four-population admixture graphs containing Mixe, Han, French, and Baka. Given the observed non-zero $f_{4}$-statistics in Table 1, there must be at least one admixture event present in order to fit the data. However, in light of the discussions above about determining which population is admixed and about parameters and constraints in admixture graphs, it would be expected that these models should be insufficiently constrained to determine which population is admixed. Indeed, they have 42=6 constraints but 2(4)+2(1)−3=7 free parameters. Confirming this expectation, perfectly fitting models (i.e., sets of branch length and mixture proportion parameters such that the six basis f-statistics are predicted exactly, yielding S(G)=0) can be obtained with Mixe specified as admixed (Fig. 3A) as well as with any of the other three populations (incorrectly) specified as admixed instead (Fig. 3B–D).

**Figure 4.**
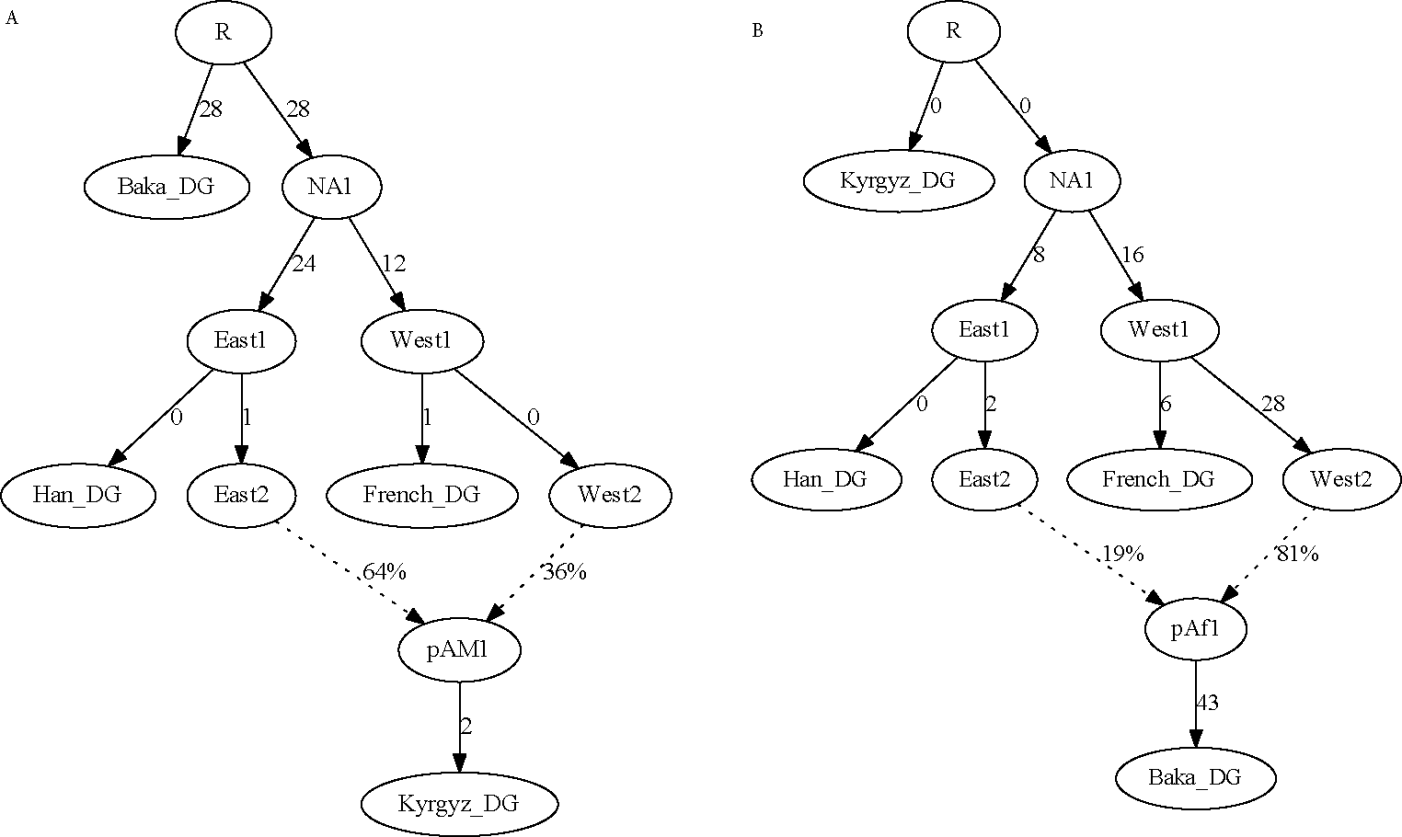
Four-population admixture graphs with Kyrgyz in place of Mixe, modeling either (A) Kyrgyz or (B) Baka as admixed. The first provides a perfect fit to the data, whereas the second has residuals up to *Z* = 27.

Interestingly, in some scenarios, the admixed population can be determined even with only four populations in the model: if a negative $f_{3}$-statistic can be formed for some triple, then the population in the first position of the statistic (i.e., population A if $f_{3}(A;\;B,\;C)\;<\;0)$ must be admixed. To give an example, I replaced Mixe with Kyrgyz in the four-population model. With Kyrgyz modeled as admixed, the fit is perfect as before (Fig. 4A). With Baka modeled as admixed, however, the fit is very poor, with residuals up to Z=27 (Fig. 4B). The most extreme residual is the statistic $f_{3}$ (Kyrgyz; Han, French), which has an observed value of −0.0064 (Z=27 for difference from zero) and can only be negative if Kyrgyz is admixed (i.e., in the position of the test population in a “three-population test” for admixture (Reich et al., 2009; Patterson et al., 2012)).

Another note is that in these examples, I have been focusing on the primary signal of deep eastern/western Eurasian admixture in Mixe. The other populations are also admixed in their own ways; for example, all of the non-Africans have small proportions of Neanderthal ancestry, and Baka are admixed with ancestry related to nearby Bantu-speaking farmers (Fan et al., 2019). However, the first signal is not evident in the data without deeper outgroups present, and the second without other African populations. Conversely, if the model contained several sub-Saharan African populations plus Mixe as the lone non-Africans, then the primary signal in our examples here would not be visible. In some ways, this inability to detect certain admixture events is beneficial, as it means that models can be constructed so as to focus on events of interest while ignoring some that are outside the desired scope of the work.

### Five populations

In general, in order to be able to solve for the parameters of an admixture graph including one admixture event, it is necessary to use at least five populations, providing n2=10 constraints for the 2n+2a−3=9 free parameters. Concurrently, in contrast to the four-population examples above, having five populations present allows one to determine which of the populations is admixed, as long as the topological relationships of the populations are all unique relative to the true mixing sources. More detail on this last point can be found elsewhere (Pease and Hahn, 2015; Lipson and Reich, 2017). A simple version of this statement is that, at least in the case of a single admixture event, one four-population subset will be unadmixed, whereas the other four subsets will include the admixed population. Similarly, in order to solve for a given mixture proportion in a larger graph, there must four populations present (aside from the admixed one in question) in distinct positions, yielding a non-redundant five-population subgraph; having three populations in distinct positions allows one to detect the signal of admixture but not to determine the proportion uniquely.

**Figure 5.**
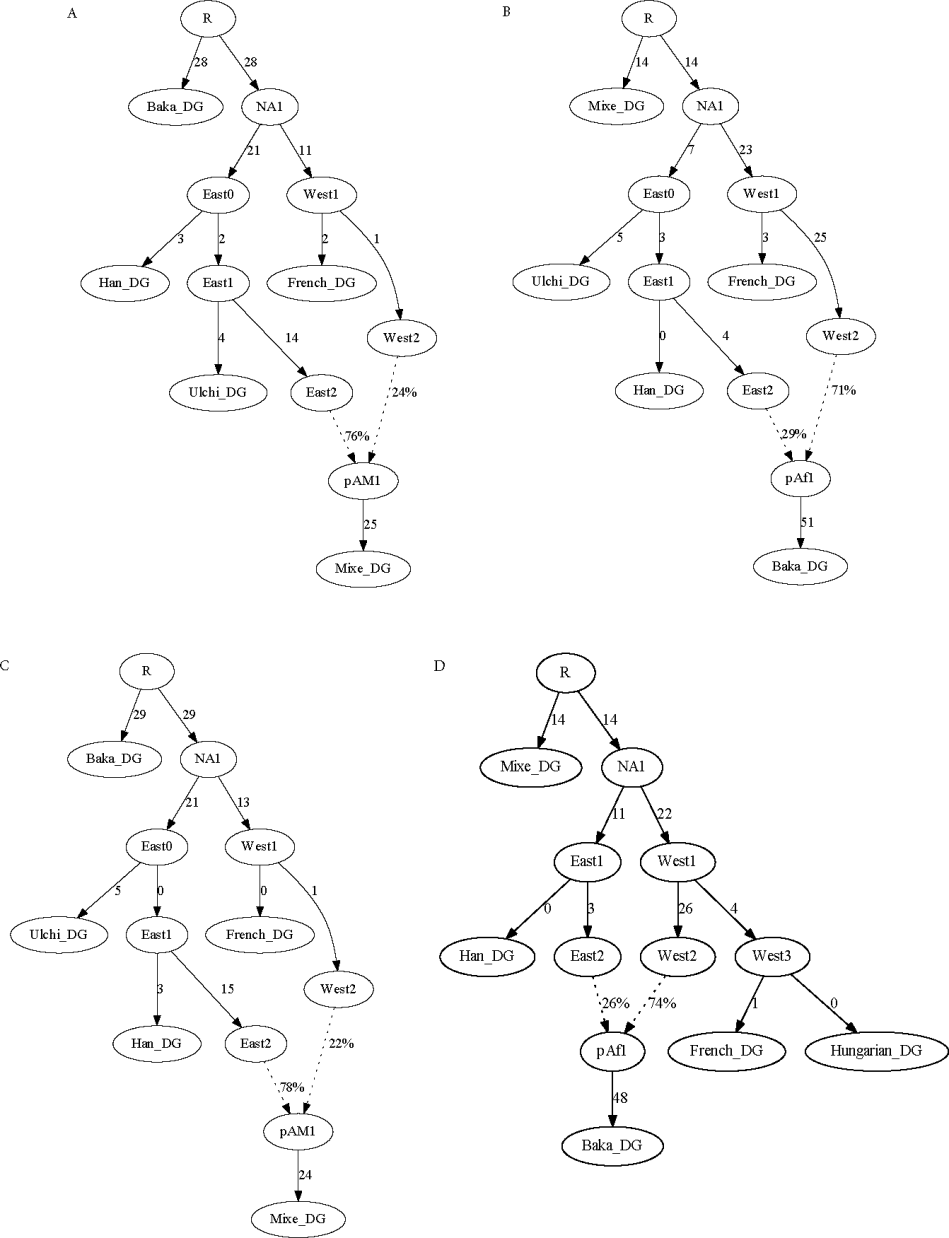
Five-population admixture graphs. (A) Standard four-population example plus Ulchi; all f-statistics are predicted to within 1.9 standard errors of their observed values. (B) Same five populations, but with Baka modeled as admixed; residual statistics are present up to *Z* = 4.7 (C) Same five populations, with Mixe modeled as admixed, but with the positions of Han and Ulchi reversed; residual statistics are present up to *Z* = 5.7. (D) Original four populations plus Hungarian, with Baka modeled as admixed; all f-statistics are predicted to within 1.2 standard errors of their observed values.

As an example, I added Ulchi (from the Amur River Basin of northeastern Asia) as a fifth population alongside the four from above. Ulchi splits closer to the eastern Eurasian source population for Mixe than does Han, which provides the additional degree of constraint. The five-population model is a good fit to the data, but not a perfect one (*Z* = 1.9 for the most significant residual; Fig. 5A). By contrast, if Baka are modeled as admixed instead of Mixe, the fit is poor (*Z* = 4.7; Fig. 5B). I also show an example where the topology is incorrectly specified, with Han closer than Ulchi to the eastern Eurasian source population for Mixe (Fig. 5C); this version fits poorly (*Z* = 5.7), and the branch connecting the split positions of Ulchi and Han collapses to length zero. If I add a second admixture event into the models in Figs. 5A–B, this creates more free parameters (11) than constraints, and indeed there are choices of the parameters that yield perfect fits, even with Mixe modeled as unadmixed (not shown).

**Figure 6.**
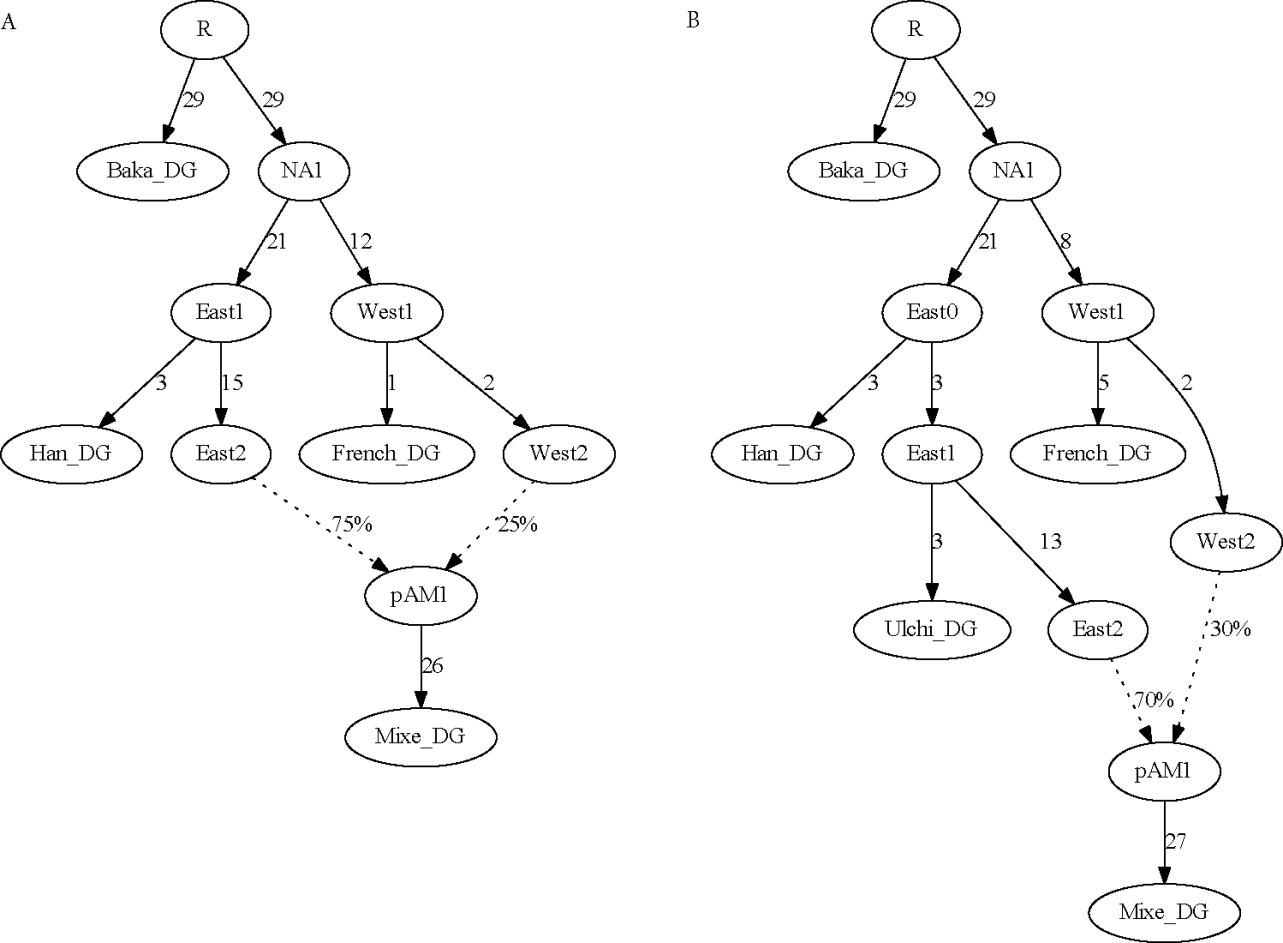
Admixture graphs with pre-specified mixture proportion parameters. (A) Four-population model, with the proportion locked at 75%; the fit is perfect. Note that the branch lengths shift slightly relative to Fig. 3A. (B) Five-population model, with the proportion locked at 70%; residual statistics (indicating a need for more eastern Eurasian ancestry in Mixe) are present up to *Z* = 2.6.

Having five populations present (with a single admixture event) also provides the ability to infer uniquely optimal parameter values. In the four-population example model, the initial estimate of eastern Eurasian ancestry in Mixe was 71%, but with the proportion manually set at 75%, the fit is still perfect (Fig. 6A). Outside of a certain range of mixture proportions (dependent on the values of the branch lengths), the fit will become worse, but within a finite interval, the likelihood is entirely flat. In terms of $f_{4}$-statistics, the observed non-zero value is being fit as equal to a branch length in the admixture graph times the mixture proportion (as in Fig. 1C), but without additional constraint, that product can remain the same while the branch length and mixture proportion covary (where the range is determined by bounds on the individual parameter values, e.g., positivity). With five populations, however, there is a unique optimal solution; for example, if I set the mixture proportion at 70% eastern Eurasian ancestry (as compared to the point estimate of 76% in the five-population model), there are residuals up to *Z* = 2.6 (Fig. 6B), and the score is more than 10 units worse. Even in the example above with Kyrgyz (i.e., a four-population model where the admixed population can be determined because of a negative $f_{3}$-statistic; Fig. 4), the parameters remain not uniquely determined.

Finally, in Fig. 5D, I show a model with the original four populations plus Hungarian instead of Ulchi. Although there are five populations present, French and Hungarian can be modeled as sister groups, so equations relating parameters in the graph to statistics of the form $f_{2}$ (French, X) and $f_{2}$ (Hungarian, X) are linearly dependent (up to their terminal branch lengths) and hence do not contribute fully independent constraints. This can be seen in the results, as Baka can successfully be modeled as the admixed population (with residuals up to *Z* = 1.2 reflecting small observed asymmetries between French and Hungarian). This contrasts with Ulchi, which has a distinct phylogenetic position from Han (relative to the other populations in the model) and thus adds new constraints (although it is worth noting again that a population with only a slightly different position adds constraint but only weakly).

## Discussion

Most of the material in this paper pertaining to admixture graphs has been presented from the perspective of the *qpGraph* software, but other methods are also available, using both different kinds of data and different fitting schemes. At the level of mathematical formulation, the results have assumed that models are fit based on a distance metric (specifically, f-statistics). As an alternative example, the *TreeMix* algorithm (Pickrell and Pritchard, 2012) is based on a maximum-likelihood framework in terms of allele frequency covariances, although the information captured is the same; see Peter (2016) for the equivalence and a thorough exploration of alternative interpretations of f-statistics in terms of population genetic models. There are also methods that use richer summaries of the data (for example, the full joint allele frequency spectrum) to infer more complicated demographic models that are similar in form, or in some cases essentially identical, to admixture graphs—for example, *∂*a*∂*i (Gutenkunst et al., 2009), G-PhoCS (Gronau et al., 2011), fastsimcoal2 (Excoffier et al., 2013), and momi2 (Kamm et al., 2019). The mathematical underpinnings of such methods are quite different from those based on f-statistics, and so the results presented here do not pertain to them. The choice of which program to use can depend on aspects of the particular application such as the data set (e.g., number of populations, whole-genome sequencing versus genotyping array, etc.) and the desired level of complexity and parametrization. Even more generally, of course, numerous other approaches exist to model population genetic structure beyond phylogenetic trees with gene flow. While it may sometimes be possible to evaluate empirically the suitability of an admixture graph for a given problem—for example, by exploring whether any graph of a reasonable size provides a good fit to the data—the choice of model is ultimately at the discretion of the analyst.

Within the class of f-statistic-based (or equivalent) admixture graph methods, there are different approaches to automation and the selection of which populations to model as admixed. *qpGraph* leaves the choice of how many admixture events to include (and which populations are admixed) up to the user; some guidelines pertaining to this choice have been discussed above. For smaller models, it can also be possible to search some or all of the full graph space (Shinde et al., 2019) to determine best-fitting topologies for a given number of admixture events (for example, using the similar *admixturegraph* R implementation (Leppälä et al., 2017) and AdmixtureBayes (Nielsen, 2018); other techniques are the subject of ongoing work). *MixMapper* (Lipson et al., 2013) provides an intermediate level of automation by attempting to infer an unadmixed sub-model and then fitting one or two admixed populations onto this scaffold. With a small set of populations, this can sometimes be a useful approach, but it can largely be recapitulated within *qpGraph*, and the software does not support large models with more admixture events. At the most automated end of the spectrum is *TreeMix* (Pickrell and Pritchard, 2012), which only asks the user to supply the list of populations and the number of admixture events and then returns a single inferred model. The advantage of this strategy is that the program does all of the work of building the graph, which is especially useful if one has limited prior knowledge about the populations. The main drawback, in my view, is that the way the program builds the graph is by starting with an optimal mixture-free tree and then adding admixture events to account for deviations between the predictions of the tree model and the observed data. Depending on the true histories of the populations, this approach can be successful, but it can also increase the chances of falling into local optima imposed by the initial tree (especially if many populations are admixed; see (Lipson et al., 2013)). Additionally—as in other methods—the choice of how many admixture events to include, which can sometimes be difficult, is still left to the user.

In my experience, I have found f-statistics and admixture graphs to be very useful tools for learning about phylogeny and admixture. I hope that this guide will help others to get the most out of these tools in a range of real-world applications.

## Figures and Tables

**Table 1.** Observed *f*_4_-statistics (values and *Z*-scores for difference from zero) for the example populations.

Populations				*f*_4_(A, B; C, D)	

A	B	C	D	Value	*Z*-score

Mixe	Baka	Han	French	0.011	27.1
Mixe	French	Han	Baka	0.013	35.8
Mixe	Han	Baka	French	−0.0025	−8.9

## Data Availability

The data that support the findings of this study are openly available through the European Nucleotide Archive (ENA), under accession numbers PRJEB9586 and ERP010710, and at the European Genome-phenome Archive (EGA), under accession number EGAS00001001959 (Mallick et al., 2016; Fan et al., 2019).
